# Diagnosis of an *Acinetobacter pittii* from a patient in China with a multiplex PCR-based targeted gene sequencing platform of the cerebrospinal fluid: A case report with literature review

**DOI:** 10.1097/MD.0000000000031130

**Published:** 2022-10-21

**Authors:** Wenliang Feng, Xuebin Jiang, Rujiang Zhang, Zhendong Guo, Daiquan Gao

**Affiliations:** a Department of Critical Care Medicine, Fengtai Youanmen Hospital, Beijing, China; b Intensive Care Unit, Renhe Hospital, Beijing, China; c Department of Neurology, Yunnan St John’s Hospital, Yunnan, China; d Department of Science and Technology, Shanghai, China; e Department of Neurology, Xuanwu Hospital of Capital Medical University, Beijing, China.

**Keywords:** *Acinetobacter pittii*, case report, metagenomic next-generation sequencing, Multiplex PCR, purulent meningoencephalitis

## Abstract

The traditional diagnosis model has great challenges for the etiological diagnosis of the central nervous system (CNS) diseases with similar clinical manifestations, especially for the diagnosis of rare pathogens. It is very important to make rapid and accurate identification of pathogens for guiding clinical choices in administering countermeasures.

**Case summary:**

On August 22, 2020, a 49 years old Chinese male patient had a headache for two days, and then the computed tomography (CT) scan of the brain showed subarachnoid hemorrhage. Subsequently, he underwent twice craniotomy and about 3 weeks of hospitalization. Since September 20, the patient was in the local rehabilitation hospital for hyperbaric oxygen therapy for about three weeks. Then the patient developed acute purulent meningoencephalitis. In the absence of diagnosis of specific pathogenic bacteria, vancomycin (1 g every 12 hours), ceftazidime (2 g every 8 hours), mannitol dehydration (125 mL, every 8 hours), and sodium valproate (0.4 g tid) was used timely according to cerebrospinal fluid (CSF) examination and clinical manifestations. CSF smear and routine culture test were negative during hospitalization. We used the metagenomic next-generation sequencing (mNGS) analysis of CSF for quick and accurate diagnosis, which identified human herpesvirus type 4 (EBV), *Corynebacterium corynebacterium*, Achromobacter xylose oxidation, and *Acinetobacter baumannii*, But the mapping degree was not high. Then, we used the modified method-multiplex PCR-based targeted gene sequencing platform (ptNGS) to detect CSF samples and found that the sequences detected were *Acinetobacter pittii* (*A. pittii*) and *Staphylococcus epidermidis*. *S. epidermidis* might come from skin colonization during lumbar puncture, so it was excluded from the etiological diagnosis. Therefore, we highly suspected that *A. pittii* was the pathogen in this case. After about three weeks of hospitalization treatment, the patient’s symptoms were relieved.

**Conclusion:**

In conclusion, empirical medication before the identification of pathogens is very important. The ptNGS may be an effective method for the diagnosis of pathogens.

## 1. Introduction

Meningitis and encephalitis are the common types of central nervous system (CNS) infection and the common causes of cerebrospinal fluid (CSF) degeneration.^[1]^ Both diseases can be caused by various pathogens such as bacteria, viruses, and fungi. The existing model of diagnosing infection depends on formulating a differential diagnosis according to the patient’s history, clinical manifestations, and imaging findings, with a series of laboratory tests.^[2]^ However, the traditional diagnosis model has great challenges for the etiological diagnosis of CNS diseases with similar clinical manifestations, especially for the diagnosis of rare pathogens. Many cases of meningoencephalitis with unknown etiology still exist.^[1,3,4]^ Failure to make the diagnosis for patients with CNS diseases in time may result in the inability to give patients accurate treatment, which might bring bad outcomes to patients. Acinetobacter calcoaceticus–baumannii (Acb) complex such as *Acinetobacter pittii* are increasingly reported in clinical specimens.^[5]^

Metagenomic next-generation sequencing (mNGS) can identify bacteria, viruses, fungi, parasites, and other pathogens by sequencing the total DNA or RNA in the samples. It provides an unbiased analysis method and can identify all pathogens with known sequences in theory.^[6–9]^ Previous prospective studies have reported that mNGS of CSF obtained from patients with meningitis or encephalitis can effectively help to identify pathogens.^[2,10]^ Our research group has designed a new platform PCR-based targeted gene sequencing platform (ptNGS) for pathogen detection, which is based on NGS and multiplex PCR. The platform has the advantages of high identification efficiency, simple operation, low cost, high throughput, and strong scalability.^[11]^

Here, we report a case of meningoencephalitis after twice craniotomy. The mNGS analysis and ptNGS was used to identify the pathogen. This study has been approved by the ethics committee of the Xuanwu Hospital of Beijing city with informed consent by the patient’s family.

## 2. Case presentation

On August 22, 2020, a 49 years old Chinese male patient presented with moderate pain at the right temporal apex without any inducement during work. The next day, severe headaches recurred with repeated vomiting several times. The patient then visited the local hospital. The patient had hypertension and hyperthyroidism for more than 2 years, and he had no travel or head injury history for the preceding 2 years.

In the local hospital, the patient had a computed tomography (CT) scan of the brain, and the results showed subarachnoid hemorrhage. Then the patient transferred to Chengde Medical College for treatment. On August 24, he underwent clipping of multiple intracranial aneurysms (middle cerebral artery and anterior communicating artery aneurysm) under general anesthesia. On the third day after the operation, the CT scan of the brain showed that there was a new hematoma in the operation area, and the temporal lobe intracerebral hematoma removal surgery through original incision was performed in an emergency. Subsequently, the patient was hospitalized for about 3 weeks. On September 20, the patient was discharged to the local rehabilitation hospital for hyperbaric oxygen therapy. In the early morning of October 10, the patient had intolerable, persistent, and severe head pain with a body temperature of 39.5 °C and non-jet vomiting for 3 to 4 times in the rehabilitation hospital. Three hours later, the patient developed limb twitch and exhalation refractory condition, lasting for 2 to 3 minutes, then the attack was terminated automatically. After the attack, the patient’s consciousness was blurred, and then recovered after about 5‐6 hours. After waking up, he complained of a headache as before.

Subsequently, the patient was transferred to the Affiliated Hospital of Chengde Medical College. Cerebrospinal fluid (CSF) examination showed pressure 200 mm H_2_O, WBC 4980 × 10^6^/L, neutrophil 98%, glucose < 1.11 mmol/L, and protein > 3 g/L. The next day, the patient was transferred to our emergency department. At that time, the patient was conscious and had a sharp headache, and intermittently appeared to talk to himself with the vague language content, accompanied by hallucinations. The diagnosis was considered purulent meningitis. A therapy of mannitol (125 mL/8 hours) and ceftriaxone sodium (2 g/day) was started. On October 13, the CSF examination showed that: pressure 240 mm H_2_O, WBC 75 × 10^6^/L, mononuclear cell 87%, glucose 42.30 mmol/L, protein 63 mg/dL, chlorine 123 mmol/L, IgA 1.06 mg/dL, IgM 0.4 mg/dL, and IgG 9.37 mg/dL. And the serum glucose was 2.2 mmol/L. The results of blood gas analysis, blood routine, serum immunoglobulin, myocardial enzymes, electrolytes, liver, and kidney function were normal. Cerebrospinal fluid smear examination results showed that no bacteria, acid-fast bacilli, and fungi were found; *Cryptococcus neoformans* (*C neoformans*) was not found by ink staining; the detection of cryptococcus neoformans membrane antigen was also negative.

On October 14, he was transferred to intensive care unit (ICU) of the neurology department for treatment. The results of the physical examination on admission were as follows: increased language, inattention, decreased memory, orientation and calculation ability, neck obstruction, chin four transverse fingers, positive Kirschner’s sign. The Padua score was 4 points, and the GCS score was 15 points. The location diagnosis was as follows: the positive headache and meningeal irritation signs located in the meninges; the mental disorders, cognitive decline, and seizures located in the cerebral cortex. The qualitative diagnosis of the patient was as follows: purulent meningoencephalitis, subarachnoid hemorrhage recovery period, hypertension, symptomatic epilepsy, bacterial pneumonia: diagnosis combined with chest CT, bilateral pulmonary nodules: diagnosis combined with chest CT, and history of hyperthyroidism. The diagnosis of tuberculous meningoencephalitis and viral meningoencephalitis was excluded according to the patient’s history and CSF examination results.

**Figure 1.**
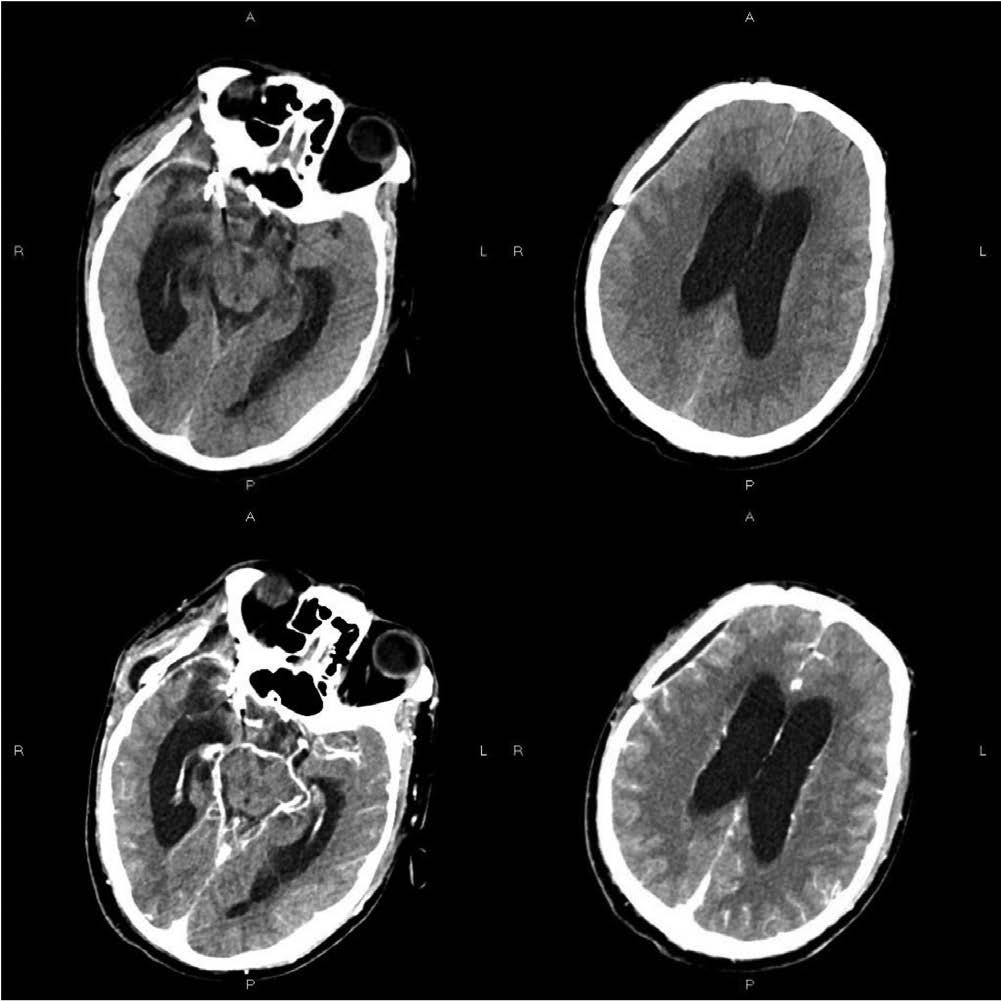
Enhanced CT scan on October 21, showed a small amount of subcutaneous effusion, subdural effusion, hydrocephalus and interstitial brain edema around bilateral lateral ventricles (A) CT scan on October 29, showed that the right temporal subcutaneous effusion and subdural effusion were less than that before. (B) Computed tomography.

Since October 14, vancomycin (1 g/12 hours) and ceftazidime (2 g/8 hours) were used for anti-infective treatment, mannitol dehydration (125 mL, every 8 hours) was used to reduce intracranial pressure, and sodium valproate (0.4 g tid) was used for antiepileptic treatment. On October 15, the relevant detection results were as follows: serum-free triiodothyronine 2.29 pg/mL, vitamin b1 2563.00 pg/mL, folic acid 6.68 ng/mL, C-reactive protein 19.80 mg/L. All tumor items, B-type natriuretic peptide, and PCT were normal. On October 17, vancomycin in serum was 10.52 ug/mL, and the patient reported that the headache weakened and the condition improved. On October 20, the patient was with low blood sodium and high intracranial pressure, then received a sodium supplement (100 mL, 10% sodium chloride), and then the situation improved. On October 21, an enhanced CT scan showed a small amount of subcutaneous effusion, subdural effusion (Fig. 1A), hydrocephalus and interstitial brain edema around bilateral lateral ventricles, and the CSF examination showed that: pressure 160 mm H_2_O, WBC 50 × 106/L, mononuclear cell 96%, fluid protein 35 mg/dL, chlorine 129 mmol/L, IgA 0.54 mg/dL. And the serum glucose was 2.52 mmol/L. The examination of cerebrospinal fluid smear, cryptococcus neoformans, and Cryptococcus neoformans membrane antigen were negative. Then the CSF sample of the patient was used for the mNGS, and the sequencing results showed that: human herpesvirus type 4 (EBV), *Corynebacterium Corynebacterium*, Achromobacter xylose oxidation, and *Acinetobacter baumannii* (*A. baumannii*) were mapped with different proportions. *Corynebacterium striatum* and *A. baumannii* were more consistent with the clinical symptoms. Then, we used the modified method-ptNGS to detect CSF samples.

Firstly, the nucleic acid of all samples was extracted by using the Zymo BIOMICS DNA/RNA Miniprep Kit (Zymo R2002). Secondly, the high-throughput sequencing library was constructed by using the Pathogeno One Pan-infectious Pathogen High-throughput Sequencing Library Construction Kit (Shanghai Pathogeno Medical Technology Co., Ltd. Cat ID Pathogeno 090118). In this process, two rounds of PCR were carried out. First, 500 microbial specific primers with clinical significance were added to the sample nucleic acid template to enrich the pathogenic target sequences; secondly, these products were purified then amplified with primers with sequencing adapter and different barcodes. Thirdly, the sequencing library was sequenced by Illumina MiSeq Reagent Nano Kit (average 0.05 M reads per library, sequencing read length = PE60). After the quality control of the data, the two-terminal alignment reading was compared with the pathogen database to determine the number of sequences (reads) in each sample.

The ptNGS results showed that the sequences detected were *A. pittii* and *Staphylococcus epidermidis*. *S. epidermidis* might come from skin colonization during lumbar puncture, so it was excluded from the etiological diagnosis. Therefore, we highly suspected that *A. pittii* was the pathogen in this case.

Five days later, the liver function test results were as follows: alanine aminotransferase 9 IU/L, total protein 61.40 g/L, albumin (bromocresol green method) 32.26 g/L, globulin 29.14 g/L, prealbumin 211 mg/L, aspartate aminotransferase 10 IU/L, creatinine (enzymatic method) 57 umol/L, urea 7.05 mmol/L, uric acid 227 umol/L, potassium 3.46 mmol/L, sodium 140.0 mmol/L, chlorine 102.0 mmol/L. The results of HBsAb, HBeAg, and HBcAg were positive.

On October 29, the CT scan showed that the right temporal subcutaneous effusion and subdural effusion were less than that before (Fig.1B). On November 2, the CSF examination showed that: pressure 145 mm H_2_O, the total number of cells 16 × 10^6^/L, cerebrospinal fluid glucose 42.48 mg/dL, cerebrospinal fluid chlorine 127 mmol/L, IgA 0.43 mg/dL. And the serum glucose was 2.36 mmol/L. Cerebrospinal fluid smear examination results showed that no bacteria, acid-fast bacilli, and fungi were found; *C. neoformans* was not found by ink staining; the detection of *C. neoformans* membrane antigen was also negative.

The results of autoimmune encephalitis related antibodies in CSF and serum samples showed that anti-NMDA receptor antibody, anti NMDA receptor antibody, or other autoimmune encephalitis related antibody were all negative. The results of cerebrospinal fluid virus-related antibodies examination showed that IgG and IgM antibodies against *Toxoplasma gondii*, rubella virus, cytomegalovirus, and herpes simplex virus type I and II were negative. The results of the blood routine test and blood biochemical test were normal.

**Figure 2.**
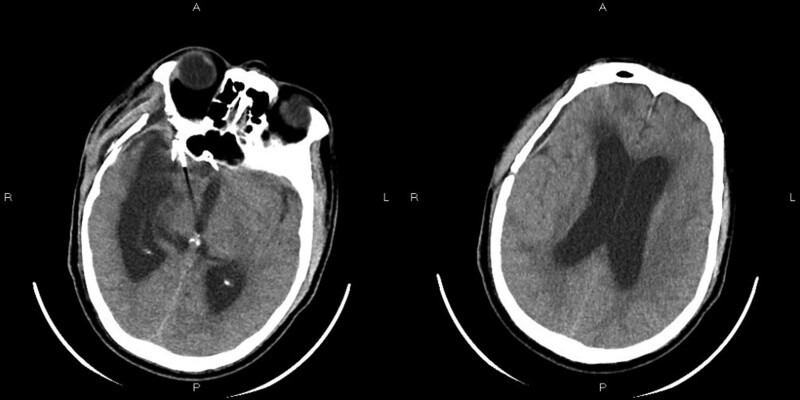
Case timeline.

On November 4, the patient’s headache symptoms almost disappeared. The results of the physical examination showed that the patients were clear in spirit and speech, indifferent in expression, inattentive in attention, decreased in memory, orientation, and calculation, negative in bilateral pathological signs, 3 transverse fingers in cervical resistance, and positive in Kirschner sign. The patient’s intracranial infection has been alleviated. He stopped the intravenous drip of antibiotics and continued anti-infection treatment with oral cefixime capsules. The patient was then discharged from the hospital (Fig. 2).

## 3. Discussion

Although intracranial infections caused by different pathogens are common, the pathogens for meningoencephalitis cases are not identified in approximately 50% of patients.^[12]^ Both empirical medications before the identification of pathogens and timely identification of pathogens have an important impact on the treatment of diseases and the prognosis of patients.

In this case, more than 40 days after craniotomy, the patient developed acute purulent meningoencephalitis. In the absence of diagnosis of specific pathogenic bacteria, vancomycin and cefotaxime were given timely treatment according to CSF examination and clinical manifestations. Cefotaxime is easy to penetrate the blood‐brain barrier and reach the focus. Vancomycin has a broad antibacterial spectrum. After a short period of treatment, the patient’s symptoms were relieved. Cerebrospinal fluid smear and routine culture test were negative during hospitalization. We then used the mNGS analysis of CSF for quick and accurate diagnosis, which identified EBV, Corynebacterium Corynebacterium, Achromobacter xylose oxidation, and *Acinetobacter baumannii* (*A. baumannii*). The number of map sequences of *A. baumannii* was only 8. But in the process of ptNGS detection, the sequences of Acinetobacter were all *A. pittii*.

As a new technology, mNGS is of great significance in the etiological diagnosis of CNS infectious diseases because it can accurately identify the pathogen.^[2,7,13–19]^ In recent years, mNGS has been successfully used to identify bacterial,^[13,14,19]^ viral,^[15,16,18]^ Ureaplasma parvum,^[17]^ toxoplasmic,^[20]^ fungal,^[21]^ and tuberculous^[10]^ pathogen in CNS infections. In a one-year, prospective, multicenter study, Michael et al reported that mNGS of cerebrospinal fluid of patients with meningitis or encephalitis improved the diagnosis of CNS infections and provided actionable information in some cases.^[2]^ In another prospective multicenter study,^[10]^ 213 patients with CNS diseases were finally enrolled, then the positive detection rate of definite CNS infections by mNGS was 57.0%. The mNGS of CSF can effectively help doctors identify pathogens of CNS diseases, which could be used and combined with traditional microbiological testing. However, mNGS also has some disadvantages. In mNGS, the specificity of detecting sequence mapping to specific species is not strong. In this case, the number of map sequences of *A. baumannii* was only 8.

ptNGS is a new platform that combines multiple PCR-based targeted gene sequencing and bioinformatics analysis of a large dataset.^[12]^ Upstream multiplex PCR enhanced the amplification of the target sequence and provided enough sensitivity. The downstream high-throughput sequencing further evaluated the count and sequence information of the amplicons. In this case, the sequences of Acinetobacter by ptNGS were all *A. pittii*, the specificity of sequence mapping to specific species was higher than the mNGS method. What’s more, in the ptNGS method, the amount of data required for a single sample library (0.01 M to ~0.03 M reads) is much lower than that of mNGS method (at least 20 M reads), which greatly improves the detection throughput and reduces the sequencing cost. Therefore, the platform has good specificity, sensitivity, cost-effectiveness, and scalability.

*A. pittii* and *A. baumannii* are the members of the Acb complex family which has emerged as an important nosocomial pathogen.^[22–24]^ *A. baumannii* has been widely studied and reported as high mortality.^[24,25]^ However, *A. pittii* is increasingly considered as the causative pathogen of nosocomial infections^[23,26]^ and is also increasing widely studied.^[22,27,28]^ But it is more and more challenging for infections with *A. pittii* because of *its* ability to develop antibiotic resistance.^[26,29–31]^ Acinetobacter infection in the CNS is relatively rare. A retrospective study showed that there were only 2 cases of CNS Acinetobacter infection in the 154 Acinetobacter infection cases, and the pathogen of those 2 cases was *A. pittii.*^[26]^ Hélène^[5]^ reported a case of New Delhi metallo-β-lactamase (NDM)-1-producing *A. pittii*, in a patient with likely cerebral abscesses. The patient got well under a combination of ceftriaxone and trimethoprim/sulfamethoxazole for 6 weeks. Fernando^[32]^ reported a case of Acinetobacter M15274 in a patient with acute encephalitis. The patient received a variety of treatment regimens, including trimethoprim/sulfamethoxazole, amikacin, and ciprofloxacin, leading to a clinical and microbiological cure. In this study, the *A. pittii* was detected by the ptNGS. After a combination of vancomycin, ceftazidime, mannitol dehydration, and sodium valproate for about three weeks, the patient evolved favorably.

## 4. Conclusion

In conclusion, empirical medication before the identification of pathogens is very important. The ptNGS may be an effective method for the diagnosis of pathogens.

## Author contributions

**Writing – original draft:** Daiquan Gao, Rujiang Zhang, Wenliang Feng, Xuebin Jiang, Zhendong Guo.

**Writing – review & editing:** Daiquan Gao.
